# Effect of bromelain on ischemia-reperfusion injury in the torsion model created in polycystic and normal ovarian tissues

**DOI:** 10.3389/fphar.2024.1451592

**Published:** 2025-01-03

**Authors:** Sevgi Ulusoy Tangul, Taylan Onat, Demet Aydoğan Kirmizi, Zuleyha Doganyigit, Emin Kaymak, Aslı Oflamaz, Atilla Şenayli, Salih Somuncu

**Affiliations:** ^1^ Department of Pediatric Surgery, Faculty of Medicine, Yozgat Bozok University, Yozgat, Türkiye; ^2^ Department of Obstetrics and Gynecology, Sincan Education and Research Hospital, Ankara, Türkiye; ^3^ Department of Obstetrics and Gynecology, Ozel Saglık Hospital, İzmir, Türkiye; ^4^ Department of Histology-Embryology, Faculty of Medicine, Yozgat Bozok University, Yozgat, Türkiye; ^5^ Department of Pediatric Surgery, Medicana Ataşehir Hospital, İstanbul, Türkiye

**Keywords:** bromelain, ischemia-reperfusion, ovarian torsion, polycystic ovary, tunel

## Abstract

**Purpose:**

Due to its increased volume, polycystic ovarian tissue is more prone to torsion than normal ovarian tissue. In treating ovarian torsion, detorsion is applied to ensure oxygenation of hypoxic tissues. However, the resulting oxygen radicals cause tissue damage. Bromelain is a substance obtained from pineapple, and studies in the literature show it is used as an antioxidant. This study aimed to evaluate the damage caused by ischemia-reperfusion (I/R) in the torsion-detorsion model created in normal and polycystic ovarian tissue and investigate the role of bromelain in this damage.

**Methods:**

Polycystic ovarian tissue was created by applying dihydroepiandrosterone sulfate to rats. Afterward, a torsion-detorsion model was used for all rats. The rats were divided into six groups: the polycystic ovary sham-operated group (P-S), the normal ovary sham-operated group (N-S), the polycystic ovary ischemia/reperfusion group (P-IR), the normal ovary ischemia/reperfusion group (N-IR), the polycystic ovary ischemia/reperfusion group treated with bromelain (P-IRB), and the normal ovary ischemia/reperfusion group treated with bromelain (N-IRB). After the procedure, tissues were collected for histopathological examination, and MDA, TUNEL, and NF-κB levels were measured.

**Results:**

This study detected significant decreases in MDA and NF-κB levels and apoptotic cell numbers assessed by TUNEL staining in groups with IR damage and given bromelain compared to the control groups. The number of TUNEL-positive cells was found to be highest in the P-IR group (8.80 ± 2.98) and significantly lower in the bromelain-administered P-IRB (1.04 ± 1.09) and N-IRB (0.52 ± 0.58) groups (p< 0.05). NF-κB expression was also high in P-IR and N-IR groups, while it was significantly decreased in bromelain-treated groups (P-IRB and N-IRB) (p< 0.05). In addition, IR damage was more pronounced in polycystic ovary tissue than in normal ovary tissue.

**Conclusion:**

Ischemia perfusion damage may be more pronounced in polycystic ovarian tissue than in normal ovarian tissue. Bromelain may be preferred to prevent I/R injury caused by ovarian torsion. It is also thought that bromelain may function in treating polycystic ovaries, and further studies can be conducted on this subject.

## 1 Introduction

In ovarian torsion, as in the mechanism of other torsions, reduced venous return and impaired arterial perfusion develop stromal edema, ovarian enlargement, and, as a result, local hemorrhage, infarction, and necrosis (Asfour et al., 2015). Surgery must be performed to restore blood flow to the adnexal area and limit damage to the ovary and the tube (Tasset et al., 2019). By detorsion, hypoxic tissues are oxygenated, forming oxygen radicals and causing tissue damage (Slater, 1988). Increased oxygen radical production and antioxidant consumption disrupt the oxidative antioxidative balance in favor of oxidative stress (Yildirim et al., 2016). To support the antioxidative course, many agents were experimentally used for ischemia-reperfusion injuries in ovary diseases (Gokalp et al., 2017; Kirmizi et al., 2021). For example, carotenoids, which are consumed via the diet, are effective antioxidants. Lycopene is one of the 600 carotenoids (Kirmizi et al., 2021). Hydrogen-rich saline solutions are also among the most commonly used antioxidants for reversing ovarian ischemia‒reperfusion. Selenium, vitamin C, protein, erythropoietin, vardenafil, and curcumin are other antioxidants used in treating ovarian torsion (Gokalp et al., 2017).

**FIGURE 1:**
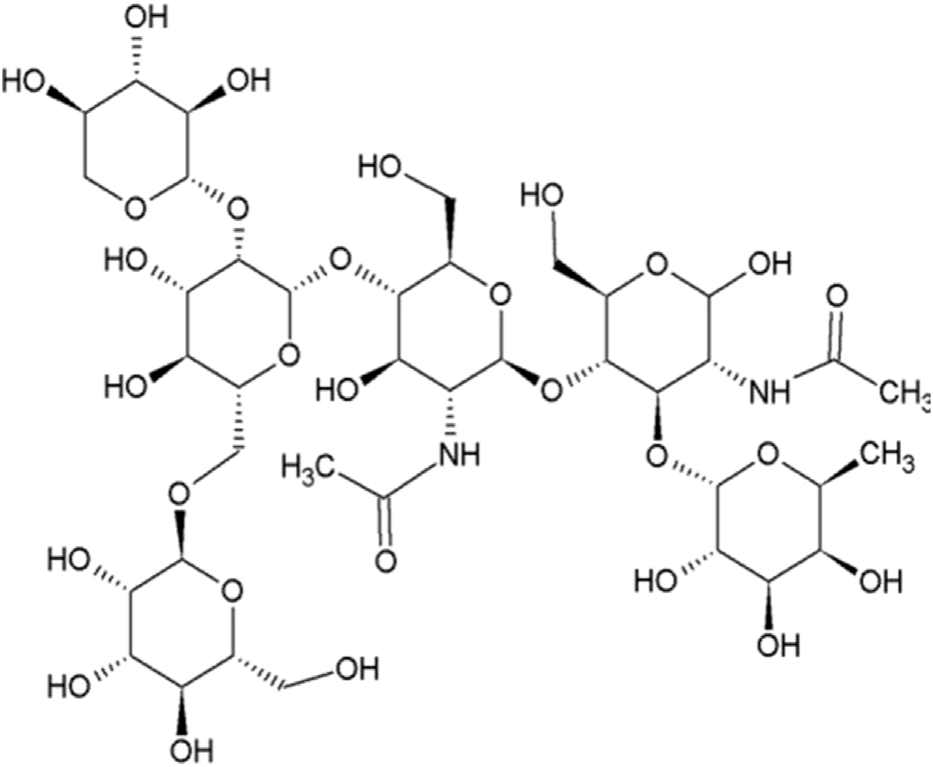
Chemical structure of bromelain (The chemical structure has been drawn by ChemDraw Professional software).

Root bromelain derived from pineapple has recently attracted significant clinical interest (Dave, 2012). IUPAC name for Bromelain is N-[(2S,3R,4R,5S,6R) 2-[(2R,3S,4R,5R)-5-acetamido-6-hydroxy-2-(hydroxymethyl)-4-[(2S,3S,4R,5S,6S) 3,4,5trihydroxy-6-methyloxan-2-yl]oxyoxan-3-yl]oxy-5-[(2S,3S,4S,5S,6R)-4,5-dihydroxy 6[[(2S,3S,4S,5S,6R)-3,4,5-trihydroxy-6-(hydroxymethyl)oxan-2-yl]oxymethyl]-3 [(2S,3R,4S,5R)-3,4,5-trihydroxyoxan-2-yl]oxyoxan-2-yl]oxy-4-hydroxy-6 (hydroxymethyl)oxan-3-yl]acetamide (Hikal et al., 2021). The chemical structure has been drawn by ChemDraw Professional software (Figure 1). Studies in the literature have shown the effect of bromelain on ischemia‒reperfusion (Bahde et al., 2007). A study by Gheisari et al. suggests that the effects of bromelain in reducing oxygen radical and inflammatory mediators may significantly reduce the damage caused by ischemia‒reperfusion injury, especially in organs such as the heart, brain, or kidneys (Gheisari et al., 2024). In their study, Po-An Hu et al. showed that Bromelain helps regulate this response by acting as an immunomodulator, balancing proinflammatory and anti-inflammatory signals to reduce tissue swelling, edema, and cell infiltration during reperfusion (Hu et al., 2022). The ability of root bromelain to reduce apoptosis and free radical formation in macrophages, its antimycobacterial properties, and its effect on foamy macrophages have been demonstrated in the literature (Mahajan et al., 2012). In a study conducted in a myocardial ischemia‒reperfusion model, the ability of bromelain to limit myocardial damage was investigated, and it was found that it improved left ventricular function more during reperfusion than did the control treatment (Juhasz et al., 2008). The literature has also shown that low doses of bromelain reduce I/R damage in the liver (Bahde et al., 2007). In the literature, bromelain is also used to treat ovarian tumors (Gani et al., 2015). However, no previous study has evaluated its effect on O/R injury.

Due to its increased volume, polycystic ovarian tissue (PCOT) is more prone to torsion than healthy tissue is, so ischemia perfusion injury may be more severe in polycystic ovarian tissue than in normal ovarian tissue. This study aimed to evaluate the damage caused by ischemia–reperfusion in polycystic ovaries and to investigate the role of bromelain in this damage. For this purpose, H&E staining was performed for histopathological evaluation, tissue MDA levels, NF-κβ values to evaluate inflammation, and TUNEL examination to evaluate cell apoptosis.

## 2 Materials and methods

### 2.1 Ethics

The experimental procedures used in this study were approved by the Kobay D.H.L. A.S Local Ethics Committee (21.02.2019 and protocol number 344. The title of the research proposal: Evaluation of ischemia-reperfusion injury in the polycystic ovary and normal ovarian tissue.). All experiments were performed by the Guide for the Care and Use of Laboratory Animals, approved by the National Institute of Health (United States).

### 2.2 Animals

In the present study, 48 female Wistar albino rats aged 2–3 months and weighing between 200 and 250 g were used. The rats were placed in stainless steel cages until the experiment was convenient at ambient temperature (24°C–25°C) and under a humid environment (55%–60%) with a controlled photoperiod (12:12 h light:dark) in standard rodent chow and water. All experimental animals were housed in cages measuring 40 cm (length) × 25 cm (width) × 20 cm (height), following the NRC Guide for the Care and Use of Laboratory Animals. Each cage contained 3 female rats, ensuring a minimum floor space of 200 cm^2^ per animal, in accordance with the recommendations for proper housing conditions and animal welfare.

### 2.3 Surgical technique

A total of 48 rats were randomly divided into six groups.

Group 1 (P-S): Polycystic ovary sham (n = 8)
Group 2 (N‒S): Normal ovary sham, (n = 8)
Group 3 (P-IR): Polycystic ovary ischemia/reperfusion (n = 8)
Group 4 (N-IR): Normal ovary ischemia/reperfusion (n = 8)
Group 5 (P-IRB): Polycystic ovary ischemia/reperfusion + bromelain (20 mg/kg intraperitoneally), (n = 8)
Group 6 (N-IRB): Normal ovary ischemia/reperfusion + bromelaine (20 mg/kg intraperitoneally). (n: 8)

#### 2.3.1 Polycystic Ovary Formation

Dihydroepiandrosthione sulfate (DHEA) was administered subcutaneously to 24 rats for 21 days at a dose of 60 mg/kg/day in the animal laboratory where the experiment was conducted, after which polycystic ovary tissue was formed. DHEA (Biosteron 25 mg; Lekam Pharmaceutical, Zakroczym, Poland) was dissolved in 0.2 mL/day of sesame oil and administered subcutaneously to rats at 60 mg/kg/day for 21 days. The PCOS pattern was proven by vaginal smear, similar to the model by Kim et al. (2018).

The rats were anesthetized using ketamine (Ketalar; Parke Davis, Eczacibasi, Istanbul, Turkey) and xylazine (Rompun, Bayer AG, Leverkusen, Germany) in combination. After anesthesia, the rats were placed in the supine position, and the lower abdomen was cleaned using 2% iodine alcohol for antisepsis. A 2.5 cm longitudinal incision was then made in the lower abdomen to visualize the right ovary.

For the sham groups (P-S and N‒S), only the abdomen was opened, and the mice were allowed to sit for 3 h. After 3 h, the ovary and the tubal tissue were removed.

To establish the ischemia‒reperfusion (I/R) groups (P-IR and N-IR), ischemia was induced using a vascular clip approximately 1 cm below the adnexal structure containing the right nasal and ovarian vessels. The incision line was closed with 4/0 nylon, and after 3 h, a relaparotomy was performed. Reperfusion was performed, and the ovaries were observed until they became pink. A reperfusion time of 3 h was applied (Kirmizi et al., 2021).

In the groups that received bromelain (PIRB and NIRB), 20 mg/kg bromelain (25 g; J&K Scientific, China) was dissolved in distilled water and then administered intraperitoneally before reperfusion (Lotz-Winter 1990).

During the surgical procedure, the vital signs of all the rats remained stable. At the end of the study, the rats were sacrificed by decapitation according to the ethics committee’s instructions. All tissues were divided into two groups: half were placed in formalin for histopathology examination, and the other half were stored at −80°C for biochemical examination.

### 2.4 Histological analysis

Ovarian samples obtained from the experimental groups were subjected to tissue tracking stages for histopathology analysis. Briefly, 5 µm thick sections obtained from tissue samples fixed in 10% formaldehyde solution and embedded in paraffin were stained with Harris hematoxylin and eosin (H&E) and examined under a light microscope (Olympus BX53) (Doğanyiğit et al., 2020). Histopathological damage scoring was performed according to the scoring system of Akdemir et al. for ovarian degeneration of follicles in the cortical area (cellular dispersion and degeneration of follicular cells), vascular occlusion, bleeding, edema, and infiltration of inflammatory cells (Akdemir et al., 2014). Each criterion was scored as usual (0), mild (Asfour et al., 2015), moderate (Tasset et al., 2019), or severe (Slater, 1988). All the results were statistically analyzed as the mean ± SD.

### 2.5 Immunohistochemical analysis

The immunoreactivity of the NF-κβ (Cat: BT-MCA 1291, Bioassay Technology Laboratory, China) protein was determined in ovary samples from the experimental groups using the avidin-biotin peroxidase method (Doğanyiğit et al., 2020; Okan et al., 2023). For this process, after deparaffinization of the sections at a thickness of 5 μm, citrate buffer was used to open the epitopes (pH = 6.0). The slides were then placed in 3% hydrogen peroxide solution in methanol to prevent endogenous peroxidase activity. Ultra V block solution was applied to avoid nonspecific staining. Subsequently, the sections were incubated with an anti-NF-κβ p65 (phospho S536) primary antibody (at: MCA 1292, Bioassay Technology Laboratory, China) at 4°C overnight. Biotinylated secondary streptavidin-HRP (TP-125-HL Lot: PHL181115; Thermo Scientific, United States) and DAB (TA-125-HD Lot: HD47396; Thermo Scientific, United States) were used as reagents, and the sections were counterstained with Gill hematoxylin. The samples were dehydrated by passing through an increasing alcohol series and covered with a sealer called Stellan. The sections were then examined with an Olympus BX53 light microscope. The immune reactivity levels were evaluated with the ImageJ program. Ten different areas were evaluated for each slide.

In order to quantify the immunohistological staining for each protein, TIFF images were imported to ImageJ/Fiji software and threshold function applied in order to separate the signal from the background and the mean signal intensity was measured by the “measure” function. The staining intensity level value was calculated by dividing the mean signal intensity above the background for a minimum 10 images per rat.

### 2.6 Apoptosis (TUNEL)

The number of apoptotic cells in the ovary sections taken from the subjects was determined using the Roche *In Situ* Cell Detection Apoptosis Fluorescein Kit (Roche-11684795910) (Doğanyiğit et al., 2020). Staining was performed according to the kit procedure. Ovary sections at a thickness of 5 µm were deparaffinized, rehydrated and washed twice with PBS for 5 min. Then, the samples were incubated at 270°C in a microwave oven in 0.01 M 5% sodium citrate buffer for antigen recovery for 5 min and then allowed to cool at room temperature for 10 min. The tissues were washed with PBS for 3 × 5 minutes and then placed in a moist chamber at 37°C. The TUNEL reaction mixture was removed from the kit and incubated in an oven for 75 min. The tissues were washed twice for 5 min with PBS and contrasted with 4′,6-diamidino-2-phenylindole (DAPI). Tissues sealed with glycerol closure solution were visualized on an Olympus BX51 fluorescence microscope at wavelengths ranging from 450 to 500 nm. For the apoptotic index, cells in 10 different areas were counted with a ×40 objective from each section.

### 2.7 Biochemical analysis

The subjects’ ovarian tissues were brought to −80°C. MDA levels in the ovarian tissue were examined. Malondialdehyde (MDA) (Cat. No: 201–11-0157; Sun Red Biological Technology) kit was used to determine MDA levels. In the analysis performed according to the working procedure of the relevant kit, the tissue samples were homogenized and then centrifuged at 2000–3,000 rpm for 20 min. The supernatants obtained were transferred to Eppendorf tubes, and the concentrations were determined as nmol/mL at 450 nm in the ELISA reader. The concentrations were then converted to nmol/mg.

### 2.8 Statistical analysis

The Kolmogorov-Smirnov test was used in this study to check whether the data were normally distributed due to small sample sizes. One‐way analysis of variance and *post hoc* Tukey tests were used to determine differences between groups. The results are presented as the mean ± SEM. The SPSS/PC program (version 20.0; SPSS, Chicago, IL) and GraphPad Prism 8.0 software were used for statistical analysis. P < 0.05 was considered to indicate statistical significance.

## 3 Results

### 3.1 Histological findings

**FIGURE 2:**
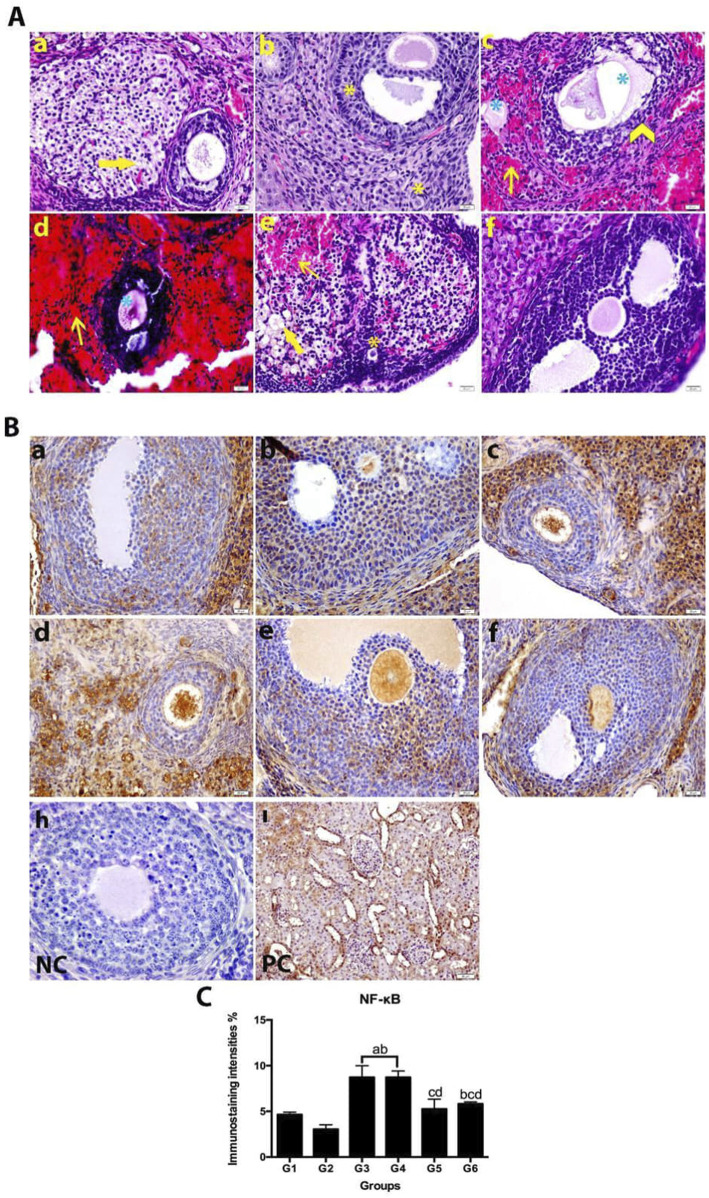
**(A)** H&E staining images of ovary tissues belonging to experimental groups. Group P-S (a), Group N‒S (b), Group P-IR (c), Group N-IR (d), Group P-IRB (e), Group N-IRB (f), magnification × 40, bar = 20 µm (Yellow arrow: vacuolization seen in cytoplasm granulosa lutein cells; yellow star: healthy ovarian follicles; blue star: atretic follicles; yellow thin arrow: vascular congestion and bleeding). **(B)** NF-κB immune staining images in ovarian tissue samples. **(C)** The data shown in the histogram graph showing the intensity of NF-κB immune-reactivity are the mean. Expressed as ±SEM. The One-way ANOVA of variance and the TUKEY *post hoc* multiple comparison test were applied (a *P* < 0.05 different to Group P-S; b *P* < 0.05 different to Group N‒S; c *P* < 0.05 different to Group P-IR; d *P* < 0.05 different to Group N-IR and e *P* < 0.05 displays difference to Group P-IRB). P-S (Group 1), N‒S (Group2), P-IR (Group3), N-IR (Group4), P-IRB (Group5), N-IRB (Group6).

While normal growing follicles were observed in group 1, atretic follicles were found in group 3. In addition, degenerated oocytes and zona pellusida were observed in the cystic follicle (Kim et al., 2018) characterized by the PCOS experimental model (Figure 2A). Using the histopathological scoring system of Akdemir et al., P-IRB and N-IRB were found to be significantly different in which ovarian tissues were treated with bromelain, PCOT or not (Table 1) (Akdemir et al., 2014). Additionally, P-IRB had a significant difference compared to N‒S and N-IR. However, the N-IRB was more important than the P-IRB was (Table 1). As shown in Figure 2A, hemorrhage was observed in and around atretic follicles in group 3. In group 4, substantial bleeding was observed in the atretic follicles and surrounding tissues. In group 5, hemorrhage and the number of degenerated follicles decreased. In group 6, antral follicles with normal histology were observed with significantly reduced bleeding. According to the histopathological data, significant improvements were observed in the ovarian tissues of the bromelain-treated groups.

**TABLE 1:** Histopathological damage scores in ovarian samples belonging to the experimental groups.

	Degeneration of follicles	Vascular congestion	Edema	Infiltration of inflammatory cells
P-S	1,5 ± 0,54	1,5 ± 0,54	1,5 ± 0,54	1,16 ± 0.41
N-S	0,5 ± 0,54^a^	0,33 ± 0,51^a^	0,5 ± 0,54	0,33 ± 0.52
P-IR	2,66 ± 0,52^ab^	2,5 ± 0,55^ab^	2,33 ± 0,82^b^	2,33 ± 0,52^ab^
N-IR	2,5 ± 0,55^ab^	2,83 ± 0,41^ab^	2,16 ± 0,75^b^	2,33 ± 0,52^ab^
P-IRB	2 ± 0,63^b^	1,66 ± 0,52^bd^	2 ± 0,63^b^	1,83 ± 0,75^b^
N-IRB	1 ± 0,63^cde^	0,83 ± 0,75^cd^	0,33 ± 0,52^acde^	0,66 ± 0,52^cde^

Data are expressed as mean ± SD. Two-way ANOVA, analysis of variance and TUKEY, *post hoc* comparison test were applied (^a^ *P* < 0.05 was different from P-S; ^b^ *P* < 0.05was different from N-S; ^c^ *P* < 0.05 was different from P-IR; ^d^ *P* < 0.05 Different from N-IR; ^e^ *P* < 0.05 represents different from P-IRB). (P-S): polycystic ovary sham, (N-S): normal ovary sham, (P-IR): Polycystic ovary ischemia/reperfusion, (N-IR): Normal ovary ischemia/reperfusion, (P-IRB): Polycystic ovary ischemia/reperfusion + Bromelain, (N-IRB): Normal ovary ischemia/reperfusion + Bromelain).

### 3.2 Immunohistochemical findings

NF-κβ expression was detected in the specimens (Figure 2B). However, the difference was insignificant between the P-IRB and N-IRB and between the P-S and N‒S (Figure 2C). NF-κβ expression was significantly lower in the P-IRB and N-IRB groups than in the P-IR and N-IR groups (*P* < 0.05) (Figure 1C). Only P-IR and N-IR (*P* > 0.05) had significantly greater expression than did the other groups (*P* < 0.05).

### 3.3 TUNEL (apoptosis) results

**FIGURE 3:**
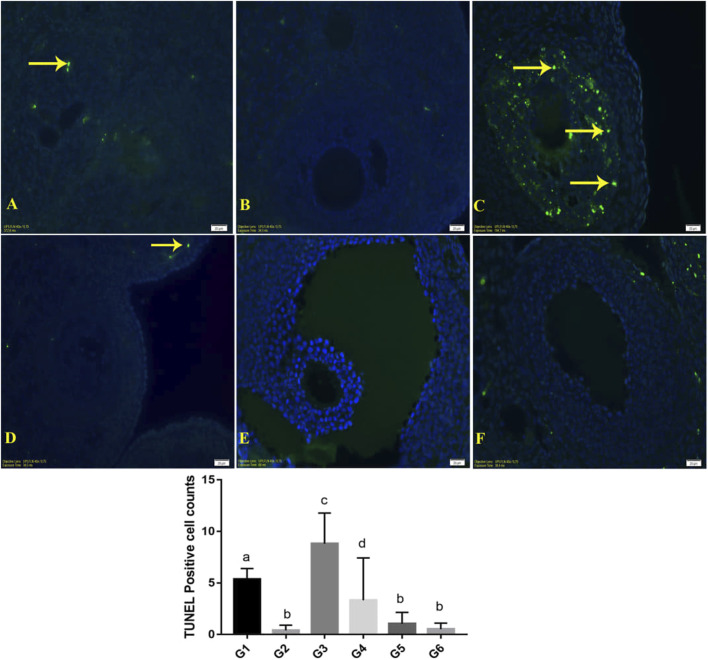
TUNEL staining images were shown. **(A)**; P-S (Group 1), **(B)**; N‒S (Group2), **(C)**; P-IR (Group3), **(D)**; N-IR (Group4), **(E)**; P-IRB (Group5), **(F)**; N-IRB (Group6). Apoptotic cells were shown with yellow arrows. X400.

Apoptosis decreased significantly in the P-IRB and N-IRB, in which bromelain was administered, according to the *P-IR* and N-IR results (Table 2; Figure 3). As expected, apoptosis was significantly greater in the P-IR group than in the P-S group.

**TABLE 2:** TUNEL positive cell numbers belonging to the groups.

Groups	P-S	N-S	P-IR	N-IR	P-IRB	N-IRB	p
TUNEL Positive cell counts	5.36 ± 1.03^a^	0.40 ± 0.50^b^	8.80 ± 2.98^c^	3.32 ± 4.11^d^	1.04 ± 1.09^b^	0.52 ± 0.58^b^	0.0001

The data are expressed as mean +standard deviation. *P* < 0.05 was accepted as significant. There were no significant differences between the groups expressed with the same letter. (P-S): polycystic ovary sham, (N-S): normal ovary sham, (P-IR): Polycystic ovary ischemia/reperfusion, (N-IR): Normal ovary ischemia/reperfusion, (P-IRB): Polycystic ovary ischemia/reperfusion + Bromelain, (N-IRB): Normal ovary ischemia/reperfusion + Bromelain).

### 3.4 Biochemical results

**FIGURE 4:**
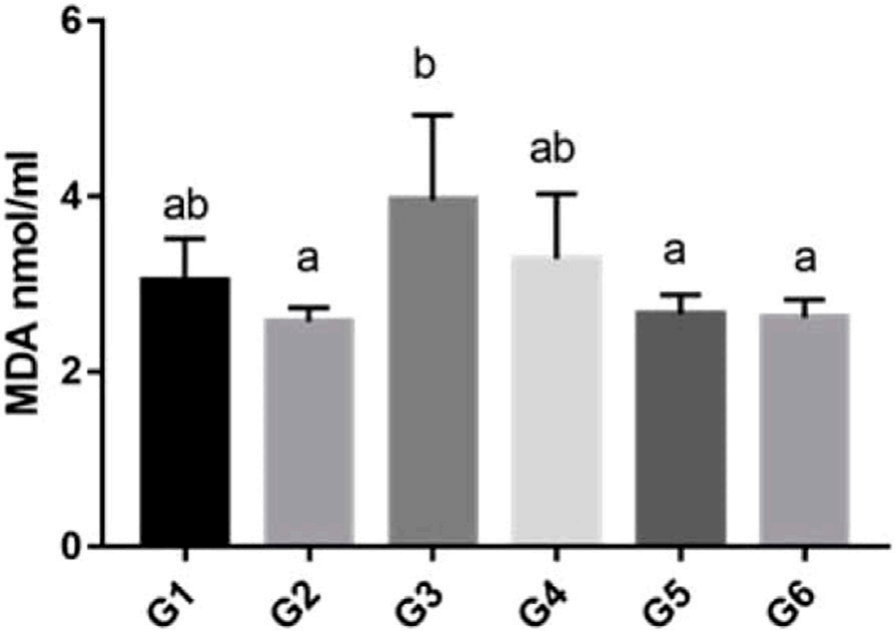
Graphics of biochemical results. p < 0.05 was considered to indicate statistical significance. There were no significant differences between the groups expressed with the same letter. P-S (Group 1), N‒S (Group2), P-IR (Group3), N-IR (Group4), P-IRB (Group5), N-IRB (Group6).

There was a statistically significant increase in MDA levels in the P-IR group compared to the N-S group. There was a statistically significant decrease in MDA levels in the P-IRB and N-IRB groups compared to the P-IR group (Table 3; Figure 4).

**TABLE 3:** Biochemical data results of the experimental groups.

Groups	P-S	N-S	P-IR	N-IR	P-IRB	N-IRB	p
MDA nmol/mg	0.000918 ± 0.000145^ab^	0,000776 ± 0,000045^a^	0,001197 ± 0,000294^b^	0,000994 ± 0,000224^ab^	0.000803 ± 0.000067^a^	0,000791 ± 0.000064^a^	0.0008

The data are expressed as mean + standard deviation. *P* < 0.05 was accepted as significant. There were no significant differences between the groups expressed with the same letter. (P-S): polycystic ovary sham, (N-S): normal ovary sham, (P-IR): Polycystic ovary ischemia/reperfusion, (N-IR): Normal ovary ischemia/reperfusion, (P-IRB): Polycystic ovary ischemia/reperfusion + Bromelain, (N-IRB): Normal ovary ischemia/reperfusion + Bromelain).

## 4 Discussion

Emerging free oxygen radicals are the main factor causing ischemia‒reperfusion -induced cell damage. Many different agents that act against these free oxygen radicals, such as antioxidant and anti-inflammatory substrates, that support the protective mechanisms of all organs have been tested experimentally, and their effectiveness has been evaluated (Gokalp et al., 2017; Kirmizi et al., 2021; Akdemir et al., 2014). In this study, we evaluated the antioxidant effects of bromelain.

Root bromelain, a widely used phytotherapeutic member of the sulfhydryl proteolytic enzyme family, is derived from Ananas comosus and has significant clinical interest (Dave, 2012). Bromelain comprises an endopeptidase, glycoprotein, and carbohydrate (Pavan et al., 2012). It has fibrinolytic, antithrombotic, and anti-inflammatory properties; these effects have been documented in animal and human studies (Juhasz et al., 2008; Pavan et al., 2012). The anti-inflammatory effect of bromelain is associated with its protease activity (Zhou et al., 2017). Furthermore, bromelain has antioxidant activity (Saptarini et al., 2019). The therapeutic effects of bromelain have been demonstrated in treating diseases such as angina pectoris, bronchitis, sinusitis, surgical trauma, thrombo-phlebitis, osteoarthritis, diarrhea, cancer, and cardiovascular disorders (Bayat et al., 2019). It has been reported in the literature that the use of recipes containing bromelain together with N-acetyl cysteine and alpha lipoic acid for the treatment of endometriosis has an anti-inflammatory effect *in vivo* and *in vitro* (Hikisz and Bernasinska-Slomczewska, 2021). Again, in a clinical study conducted on patients with endometriosis, it was stated that the use of preparations containing N-acetyl cysteine, alpha lipoic acid, and bromelain reduced the level of pain and led to decreased analgesic needs (Lete et al., 2018). Although the use and efficacy of bromelain in many tissues have been demonstrated, to our knowledge, no studies have investigated the effect of bromelain on ischemia‒reperfusion injury in patients with ovarian torsion.

In the literature, there are many studies on ovarian ischemia, edema, follicular cell damage, vascular occlusion, hemorrhage, neutrophil infiltration, histopathology evaluation, and the apoptosis index (Gokalp et al., 2017). The presence of cystic follicles, increased plasma testosterone concentration, and an increase in the number of atretic follicles have been shown in animal models of DHEA-induced PCOT compared to controls (Misugi et al., 2006). However, an ovarian torsion model created by PCOT has not been established in the literature; hence, histopathology images that may be obtained after torsion are unavailable. Nevertheless, in line with the data obtained from this study, we demonstrated that there might be less bleeding due to the scarcity of atretic follicles and the tissue around the follicles. In the present study, vacuolization was observed in the cytoplasm of granulosa lutein cells in the corpus luteum in P-S mice. In P-IR mice, bleeding in atretic follicles and surrounding tissue was observed. When bromelain was administered, the degree of bleeding decreased in the P-IR group, and vacuolization in the cytoplasm of luteal cells in the corpus luteum and healthy primordial follicles was observed.

The accumulation of abnormal cellular free oxygen radicals and I/R damage cell proteins and membranes and cause damage by activating proapoptotic pathways (Kirmizi et al., 2021). Nuclear factor kappa beta (NF-κβ) is a pleiotropic transcription factor and marker that plays an essential role in inflammatory processes. NF-κB activation occurs through radical oxygen species (ROS) and cytokines. The NF-κβ signaling pathway may increase inflammation and apoptosis (Ali et al., 2019). In this study, as expected, NF-κB expression was similar in both the N-IR and P-IR groups (*P* > 0.05) and significantly higher than that in all the other groups. However, no difference was found between normal and PCOT ovarian tissues (*P* < 0.05). Administration of bromelain significantly reduced NF-κβ (*P* < 0.05). These findings indicate that bromelain significantly reduced the degree of apoptosis caused by ovarian torsion. Our results are consistent with other results in the literature (Ali et al., 2019; Müller et al., 2016; Habashi et al., 2016).

TUNEL staining revealed that the number of apoptotic cells significantly increased in the direction of PCOT between normal and PCOT ovarian I/R tissues. Bromelain administration significantly reduced the number of apoptotic cells in both standard and PCOT ovarian tissues. Our results are consistent with other results in the literature (Zhu et al., 2022; Ikeda-Imafuku et al., 2022). Bromelain’s apoptotic effects are primarily due to its proteolytic activity through the thiol group in its structure. This activity activates apoptotic proteins, suppresses anti-apoptotic proteins, and modulates critical signaling pathways. Features such as glutamate residues in its active site and glycosylation, which increase its structural stability, optimize bromelain’s biological effects (Agostinis et al., 2015; Hikisz and Bernasinska-Slomczewska, 2021). Interestingly, bromelain significantly reduced the number of apoptotic cells in the PCOT group. These findings suggest that the use of bromelain for treating PCOT can open new horizons and that further studies can be conducted on this topic.

Reperfusion of ischemic tissue leads to more severe tissue damage than ischemia (Zimmerman and Granger, 1992). Therefore, studies aimed at preventing reperfusion injury are important (Ingec et al., 2011). Lipid peroxidation in the cell is the most harmful effect of free oxygen radicals and is accompanied by a decrease in membrane potential and subsequent cell damage. Malondialdehyde (MDA), one of the end products of lipid peroxidation, causes severe cell damage (Behroozi-Lak et al., 2017). In our study, MDA levels increased significantly in the P-IR group. There was a significant difference in the MDA levels between the N‒S and P-S groups (*P* < 0.05). The MDA levels decreased in the P-IRB, but no significant difference was found in the NI-R compared to the P-IRB. Our results for bromelain are similar to those in the literature (Ali et al., 2019; Arikan et al., 2010; Ugurel et al., 2017).

The degree of ischemic perfusion damage in polycystic ovarian tissue may be greater than that in normal ovarian tissue. However, there need to be a literature study investigating this issue. In these respects, this study is the first in the literature.

Bromelain can be used to prevent I/R injury due to PCOT-related ovarian torsion. It is also thought that bromelain may be useful for treating ovarian torsion, and further studies can be conducted on this subject. Although studies have been conducted during the literature review on the positive effects of bromelain combined with N-Acetyl Cysteine and Alpha-Lipoic Acid on endometriosis *in vivo* and *in vitro* (Agostinis et al., 2015), no study has been found on the effectiveness of bromelain in patients with PCOT or *in vivo*. From this perspective, comparative studies between the treatment methods used in PCOT patients and bromelain seem to open new horizons.

## Data Availability

The original contributions presented in the study are included in the article/supplementary material, further inquiries can be directed to the corresponding author.
